# Silencing of Reversion-Inducing Cysteine-Rich Protein with Kazal Motifs Stimulates Hyperplastic Phenotypes through Activation of Epidermal Growth Factor Receptor and Hypoxia-Inducible Factor-2α

**DOI:** 10.1371/journal.pone.0084520

**Published:** 2013-12-20

**Authors:** You Mie Lee, Sun-Hee Lee, Kheun Byeol Lee, Minh Phuong Nguyen, Min-Young Lee, Gyu Hwan Park, Mi Jeong Kwon

**Affiliations:** 1 Research Institute of Pharmaceutical Sciences, College of Pharmacy, Kyungpook National University, Daegu, Republic of Korea; 2 School of Life Sciences and Biotechnology, Kyungpook National University, Daegu, Republic of Korea; University of Hawaii Cancer Center, United States of America

## Abstract

Reversion-inducing cysteine-rich protein with Kazal motifs (RECK, a tumor suppressor) is down-regulated by the oncogenic signals and hypoxia, but the biological function of RECK in early tumorigenic hyperplastic phenotypes is largely unknown. Knockdown of *RECK* by small interfering RNA (siRECK) or hypoxia significantly promoted cell proliferation in various normal epithelial cells. Hypoxia as well as knockdown of *RECK* by siRNA increased the cell cycle progression, the levels of cyclin D1 and c-Myc, and the phosphorylation of Rb protein (p-pRb), but decreased the expression of p21^cip1^, p27^kip1^, and p16^ink4A^. HIF-2α was upregulated by knockdown of RECK, indicating HIF-2α is a downstream target of RECK. As knockdown of *RECK* induced the activation of epidermal growth factor receptor (EGFR) and treatment of an EGFR kinase inhibitor, gefitinib, suppressed HIF-2α expression induced by the silencing of RECK, we can suggest that the RECK silenicng-EGFR-HIF-2α axis might be a key molecular mechanism to induce hyperplastic phenotype of epithelial cells. It was also found that shRNA of RECK induced larger and more numerous colonies than control cells in an anchorage-independent colony formation assay. Using a xenograft assay, epithelial cells with stably transfected with shRNA of RECK formed a solid mass earlier and larger than those with control cells in nude mice. In conclusion, the suppression of RECK may promote the development of early tumorigenic hyperplastic characteristics in hypoxic stress.

## Introduction

Reversion-inducing cysteine-rich protein with Kazal motifs (RECK) is a tumor suppressing, membrane-anchored glycoprotein that contains multiple epidermal growth factor-(EGF)-like repeats and multiple serine protease inhibitor-like domains [1]. RECK inhibits proMMP-2 activation and the enzymatic activities of MMP-2, MMP-9, and membrane type 1 (MT1)-MMP [2,3]. Although RECK functions as an inhibitor of matrix metalloproteinases (MMPs), it does not share structural homology with tissue inhibitors of metalloproteinases (TIMPs) [2]. RECK is expressed in various normal human tissues, but has not been detected in oncogenically transformed cells and in various type of cancers, such as, hepatoma, pancreatic, breast, lung, colorectal, prostate, and gastric cancer, or in osteosarcomas [4]. RECK downregulation in cancer tissues is associated with a low survival rate and a poor prognosis because RECK inhibits angiogenesis, invasion, and metastasis in cancer via MMP inhibition [4]. Germ line knockout of *RECK* induced the proliferation of mouse embryonic fibroblasts (MEFs) and enabled early escape from the cellular senescence induced by oncogenic insults, and RECK interferes with epidermal growth factor receptor (EGFR) signaling [5]. Epigenetics, such as, histone and DNA modifications, are involved in the silencing of *RECK* [6,7]. We previously reported that RECK is downregulated under hypoxic conditions through HDAC1 and hypoxia inducible factor (HIF)-1α [8]. Several microRNAs are also involved in targeting RECK in hypoxia and RAS-mediating signaling pathways [9]. Thus, it has been suggested that HIF-1α is a key regulator to inhibit RECK expression in tumorigenesis [8,9]. 

Hypoxia (a reduction in tissue oxygen tension) is frequently detected in growing tumors larger than 1 mm^3^ [10]. Interestingly, in inflamed tissues, oxygen concentrations are often far below physiological levels [11]. Hypoxia results in adaptive changes to the transcriptions of a wide range of genes involved in increasing oxygen availability to tissues and decreases the cellular consumption of oxygen such as angiogenesis or glycolysis or apoptosis via HIFs-α [10]. Differential activity and expression pattern of HIF-1α and HIF-2α in the tissues have been well identified [12]. In cell cycle progression, stabilization of HIF-1α results in cell cycle arrest due to the inhibition of the transcriptional activity of c-Myc. On the other hand, HIF-2α exhibits opposing effects that is, it increases cell proliferation by activating c-Myc [13]. 

Many reports have investigated that hypoxia attenuates the expressions of tumor suppressor genes, such as, *p53, von Hippel Lindau (VHL*)*, MLH1, BRCA1, 2*, and *RUNX3* including *RECK* in normal and cancer cells [8,9,14-17]. The products of these tumor suppressor genes primarily function as gatekeepers of cell proliferation, and thus, loss of function or silencing of the tumor suppressors play critical roles in the processes that permit the unregulated proliferation and transformation of normal cells under precancerous hypoxic conditions [18-20]. However, biological functions and significances of RECK silencing under hypoxic conditions in hyperplastic phenotypes of early tumorigenesis have largely unknown. 

Here we demonstrate that RECK silencing under hypoxic conditions induced cell proliferation in normal epithelial cells and their tumorigenic potential such as anchorage-independent colony forming ability. Knock-down of RECK expression by siRNAs increased c-Myc-mediated cell cycle progression. Our results also suggest that RECK might be an upstream regulator that suppresses HIF-2α through EGFR in obtaining an early tumorigenic hyperplastic phenotype. Therefore, our data reveal a novel mechanism of *RECK* and hypoxic conditions for the induction of hyperplastic cells in an early step of tumorigenesis through HIF-2α and EGFR. 

## Materials and Methods

### Ethnics Statement and Chemicals

Animal care and experimentation were performed in accordance with procedures approved by the KNU (Kyngpook National Unversity) Animal Care and Use Committee. An inhibitor for MMP-2 and -9 (#444241), an ERK MAPK inhibitor, PD98059 were purchased from Merck Millipore, and gefitinib, an EGFR specific inhibitor was purchased from Cayman Chemical (Ann Arbor, MD). 

### Cell culture and hypoxic incubation

HEK293 human embryonic kidney and TCMK mouse kidney (American Type Culture Collection, Manassas, VA) cells were maintained in DMEM supplemented with 10% fetal bovine serum (FBS, Hyclone, Logan, UT) and 1X antibiotics (100 units/ml penicillin, 100 µg/ml streptomycin, both from Invitrogen, Carlsbad, CA). MCF10A normal human breast epithelial cells were maintained in DMEM/F12 supplemented with insulin, cholera toxin (Sigma, St Louis, MO), rEGF (R&D systems, Wiesbaden, Germany), hydrocortisone, and L-glutamine (Invitrogen, Gaithersburg, MD). PrEC prostate epithelial cells (Clonetics Cambrex Corp, Walkersville, MD) were cultured in keratinocyte growth media (Lonza, Basel, Switzerland) containing 10% FBS and 1X antibiotics. Cultures were maintained under either hypoxic (1% O_2,_ 5% CO2 and balanced with N2) or normoxic (21% O_2,_ 5% CO2 and balanced with N2) conditions at 37°C (Thermo Scientific, MA, USA). 

### Semiquantitative and real-time reverse transcription (RT)-PCR

Gene expressions were analyzed using semiquantitative and real-time RT-PCR as previously described [21]. 

### siRNA experiment and transfection

Two double strand siRNAs designed to target *RECK* (Qiagen, Valencia, CA), HIF-1α, and HIF-2α and a scrambled siRNA were synthesized (Bioneer, Daejeon, Korea). HEK293 cells were transfected with double strand RNA using HiPerFect Transfection reagent (Qiagen) according to the manufacturer's protocol. *RECK* mRNA expression was evaluated by RT-PCR and RECK protein expression was monitored by western blotting at the indicated times post-transfection. Proliferation assays were performed on 96 well after splitting cells at 24 h post-transfection. SiRNA1 was designed to target 5’-CAGATTGAAGCCTGCAATAAA-3’, and siRNA2 was designed to target 5’-ATACCTGTTCTTGATATTAAA-3’. pBluescript *RECK* full-length plasmid (Invitrogen) was cloned into pCMV5 vector after digestion with KpnI and SacI. Plasmids were transfected into HEK293 cells that had been plated at 1x10^6^ cells per 60 mm dish one day previously. Transfections were performed using Lipofectamine 2000 reagent (Invitrogen) according to the manufacturer’s instructions. At 24 h post-transfection, cells were plated onto 96-well plates for proliferation assays or onto 60 mm dishes for protein collection. 

### Western blot analysis

Equal amounts of protein extracts in SDS-lysis buffer were subjected to SDS-PAGE analysis and then transferred to nitrocellulose membranes. We used the following human antibodies: anti-RECK (BD Bioscience, San Diego, CA), cyclin D1, cyclin A, p27, NFkB (Santa Cruz Biotechnology, Santa Cruz, CA), p21 (Cell signaling), phosphorylated EGFR, ERK1/2 (Cell Signaling), EGFR (Santa Cruz Biotechnology, Santa Cruz, CA), β-actin, and α-tubulin (Sigma, St Louis, MO). An enhanced chemiluminescence system (Pierce, Woburn, MA) was used for detection. Antibody for pEGFR may cross-reacts slightly with other EGFR family member (eg. ErbB2) or activated PDGF receptor.

### Cell proliferation assay and cell cycle analysis

Cell proliferation assays were performed by using Cell Counting Kit-8 (CCK-8) solution (Dojindo, Gaithersburg, MA). Cells (5 x 10^3^) were seeded onto a 96-well plate. After 24 hours (time 0), we started to measure cell proliferation at the indicated time points. When we seeded 5 x 10^3^ cells in wells, one day later densities became around 20%. Because the cell proliferation rate in hypoxia was higher than in normoxia, only hypoxic cells were almost confluent (- 95%) after 72 h of incubation. The graph represents fold changes in numbers of cells at each time versus control cell number at time 0. Cells were plated at 1x10^5^ per 60 mm dish and exposed to normoxic or hypoxic condition for the indicated times. The DNA contents and cell cycle statuses of permeabilized cells were determined by propidium iodide (PI, Sigma-Aldrich) staining. Stained cells were processed using a fluorescence-activated cell sorter (FACS, Coulter Elite ESP Cell Sorter, Beckman) and cell cycle profiles were analyzed. 

### Soft agar colony formation assay

Soft agar colony formation assays were performed using the CytoSelect 96-well Cell Transformation Assay kit (Cell Biolabs, San Diego, CA). Ten days after seeding, the numbers and morphologies of colonies were determined using an inverted phase-contrast microscope (Olympus, Japan). To quantify anchorage-independent growth, colonies were lysed with lysis buffer and detected with CyQuant GR dye. Fluorescence was measured using a fluorometer and a 485/520 filter set (Wallac Victor3 1420 mutilabel counter, PE). Data are presented as the means ± SDs of three independent wells. 

### RECK shRNA stable transfection

To establish RECK shRNA stable transfectants, we synthesized shRECK oligonucleotides harboring the RECK siRNA1 sequence and cloned them into a blasticidine-resistant pBLOCK-iT Gateway vector (Invitrogen), according to the manufacturer's instructions. For a control vector, we used a lamin shRNA entry clone and used the same procedure used for the cloning of RECK shRNA into the destination vector. shRNA transfectants were selected by treating cells with blasticidine (5 µg/ml) for 21 days. Cells stably transfected with shRECK or shlamin were established and treated weekly with blasticidine (5 µg/ml) in DMEM containing 10% FBS. 

### Animal experiments and immunohistochemistry

Balb/c-nu mice were purchased from the Institute of Medical Science, University of Tokyo (Tokyo) and maintained in a specific pathogen free facility in accordance with the guidelines issued by the Animal Care and Use Committee of Kyngpook National University. Animals were provided with autoclaved tap water and lab chow *ad libitum* and were housed in a 23 ± 0.5°C, 55 ± 10 % relative humidity environment under a 12 hour light-dark cycle. For tumorigenesis experiments, 1x10^7^ cells stably transfected with shRECK or shlamin were suspended in 0.2 ml of Matrigel and injected into both flanks of nude mice. Tumor growths were determined by measuring the dimensions of tumors with a caliper every two days and multiplying tumor height x length x depth (mm^3^). Tumor tissues were fixed in 4% paraformaldehyde (pH 7.4) and embedded in paraffin or OCT. Serial sections (5 µm) were mounted on poly-L-lysine coated slides, and processed for either histology or immunohistochemistry. Sections were immunostained with antibodies against mouse CD31 (BD, NJ), p-pRb, PCNA, p16, CA9, RECK (Cell Signaling), and a hypoxic probe (Chemicon International), and visualized using appropriate biotin-conjugated secondary antibodies followed by immunoperoxidase detection (Vectastain ABC Elite kit; Linaris, Germany) using diamino-benzidine (DAB) as substrate (Vector, U.K). Counterstaining was performed with hematoxylin. Hypoxic probe-1 (pimonidazol HCl, Chemicon International) was injected 4 h before tumor excision. 

### Statistical analysis

ANOVA was performed to assess the significances of differences between experimental groups. Statistical significance was accepted for p values < 0.01. Results are represented as means ± SD. 

## Results

### Hypoxia stimulated proliferations of human epithelial cells

**Figure 1:**
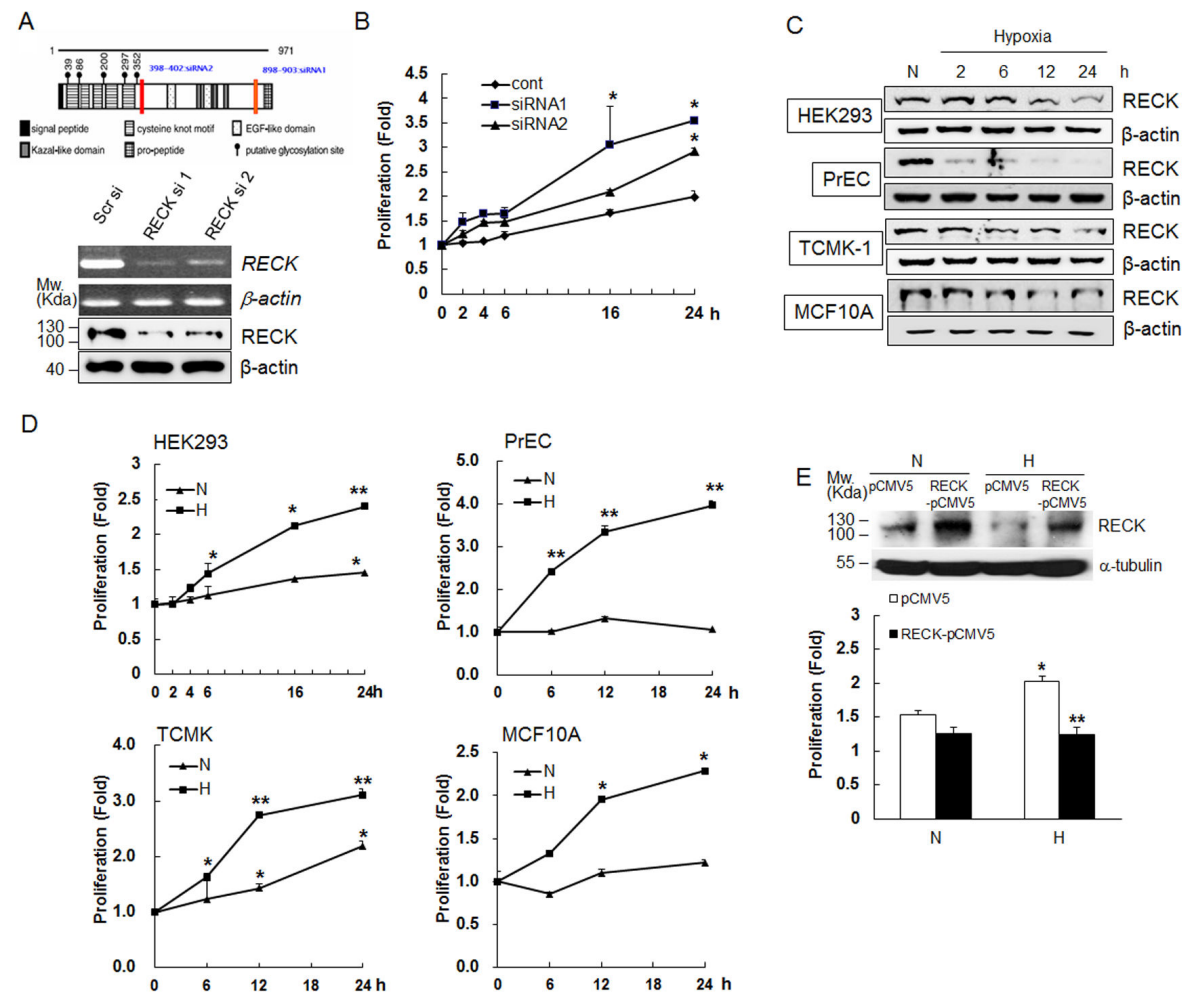
RECK silencing induced by hypoxia stimulated epithelial cell proliferation. (**A**) Site and silencing efficiency of two RECK siRNAs. The upper panel shows RECK siRNA sites (red bar). The lower panel shows that transfection of RECK siRNAs (5 nM) into HEK293 cells effectively blocked RECK expression. The experiment was performed in triplicate. (**B**) Proliferation assays were performed at 24 h post-transfection (0 time) at the indicated times after seeding siRNA-transfected cells at 5x10^3^ per well in 96-well plates. Fold change is the ratio of the cell population at each time point versus the start time (point 0). Data are presented as means ± SDs (n=4). *, *p*<0.01 versus scrambled-transfected controls. (**C**) Total protein lysates were extracted from HEK293, PrEC, TCMK, and MCF10A cells after exposure to normoxia or hypoxia for the indicated times. RECK expressions were determined by western blotting. β-actin was used as an internal control. (**D**) HEK293, PrEC, TCMK, and MCF10A cells were plated at a density of 5x10^3^ in 96-well plates. After 24 h, cells were maintained under normoxic (N) or hypoxic (H) conditions for the indicated times. Cell proliferation assays were performed using a CCK-8 cell proliferation assay kit. Data are presented as means ± SDs (n=4). *, *p*<0.01, **, p<0.001 versus normoxic controls. (**E**) Full-length *pCMV5-RECK* plasmids were transfected into HEK293 cells, and RECK protein levels and cell proliferation rate were determined by Western blot analysis and CCK-8 cell proliferation assay kit, respectively, after incubation under normoxic (N) or hypoxic (H) conditions for 24 h. Data are presented as means ± SDs (n=3). *, *p*<0.01, versus normoxic controls, **, p<0.01 versus hypoxic controls. All experiments were performed at least three times.

To identify whether the downregulation of *RECK* is involved in regulation of cellular growth or not, we blocked *RECK* expression using siRNA and performed cell proliferation assays in HEK293 kidney epithelial cells under normoxic conditions. *RECK* mRNA and protein levels were clearly diminished at 48 h after transfection with two different siRNAs (Figure 1A). Downregulation of RECK by siRNAs clearly increased cell growth with time, and cell growth rate was inversely correlated with the degree of RECK suppression by siRNA 1 (by 3.5 fold) or 2 (by 2.9 fold) (Figure 1B). 

Previously, we found that RECK was silenced at the transcriptional level by HDAC and HIF-1α in HEK 293 cells under hypoxic conditions [8]. When we examined RECK protein levels in four types of normal epithelial cells isolated from specific tissues, HEK293 (human kidney), PrEC (human prostate), TCMK (mouse kidney), and MCF10A (human breast) after exposed to hypoxia, we observed RECK expression was decreased in a time-dependent manner under hypoxic conditions (Figure 1C). Exposure to hypoxia for 6 h significantly stimulated the proliferations of HEK293, PrEC, TCMK, and MCF10A cells. As compared with cells maintained under normoxic conditions, proliferations increased by 2.0-3.2 fold after 12-16 h of hypoxia and by 2.3-4.0 fold after 24 h (Figure 1D), when proliferations maintained a steady state due to confluent cell densities. Because the cell proliferation rate in hypoxia was higher than in normoxia, only hypoxic cells were almost confluent (-95%) after 72 h of incubation. We restored *RECK* expression with a full-length *RECK-*pCMV5 plasmid, and then found that cell proliferation was significantly inhibited under hypoxia (Figure 1E). These results strongly suggest that hypoxia-induced RECK downregulation is responsible for obtaining hyperplastic properties of normal epithelial cells.

### RECK downregulation induced by hypoxia was responsible for cell proliferation

**Figure 2:**
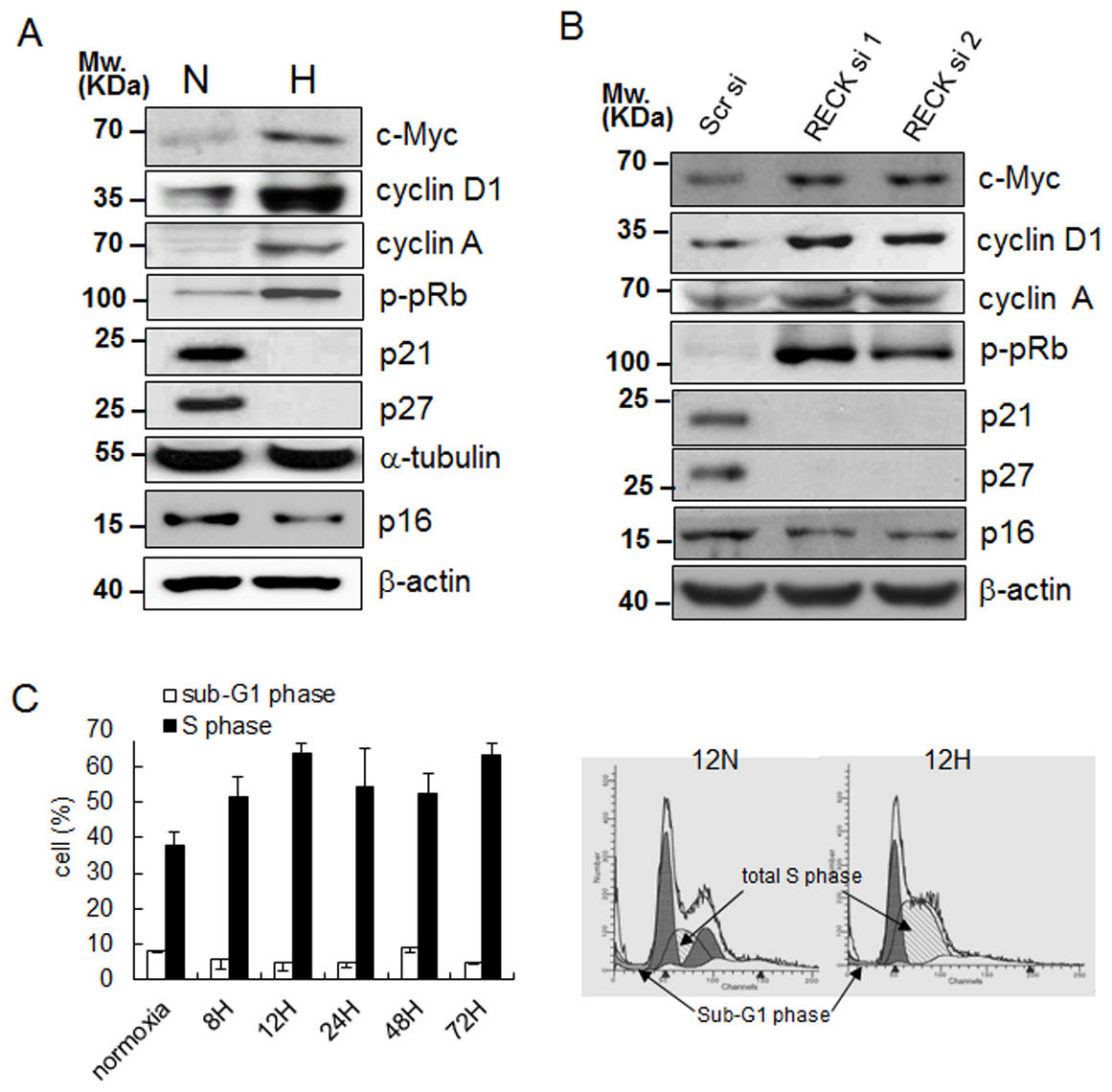
Hypoxia and silencing of *RECK* increased cell cycle progression. (**A**) Western blot analysis with anti-c-Myc, cyclin D1, cyclin A, phosphorylated Rb (p-pRb), p21, p27, and p16 were performed under normoxic (N) and hypoxic (H) conditions after 24 h of exposure. (**B**) After transfecting scrambled siRNA (Scr) and two kinds of siRECK (si1 and si2), levels of cell cycle proteins in cell lysates were determined by Western blotting. (**C**) Cell cycle analysis by FACS after PI staining. The diagram shows the diploid S and sub-G1 phase HEK293 cells under hypoxic conditions up to 72 h after staining and those of normoxic controls (cont). Right cytograms show the proportion of cell populations in each cell cycle stage in 12 h normoxia (12N) and hypoxia (12H). All experiments were performed at least in duplicate.

We next measured the levels of various cell cycle proteins under hypoxic conditions and in parallel with the silencing of RECK in HEK293 cells. Levels of cyclin D1, cyclin A, and phosphorylated-Rb (p-pRb), as well as c-Myc (a critical factor for G1-S transition) were increased both by hypoxic conditions and by the silencing of RECK (Figure 2A, B). Consistently, levels of p21^cip1^, p27^kip1^ and p16ink4A that inhibit cyclin/CDK activity at the G1-S transition point, were depressed under hypoxic conditions and by the silencing of RECK (Figure 2A, B) and these results are in-line with our observations of increased cell proliferation shown in Figure 1. These results can suggest that hypoxia-induced RECK downregulation is responsible for obtaining hyperplastic properties of normal epithelial cells. To determine if hypoxia-induced cell proliferation was due to a decrease in cell death and/or an increase in cell cycle progression, we subjected HEK293 cells to flow cytometry. Cells cultured under hypoxic conditions for 8 h to 72 h showed 1.6 fold increases in the proportions of cells in S phase as compared with cells cultured under normoxic conditions. This increase in the proportion of cells in the S phase continued (with some fluctuation) over 72 h of hypoxic exposure (Figure 2C, see the dashed area for diploid S phase in 12 h hypoxia). Cell death was monitored by counting subG1 phase populations, and the measurements obtained suggested that hypoxia did not induce cell death (Figure 2C). These results suggest that an increase the S-phase transition and a lack of change in cell death collaborate to stimulate cell viability and proliferation under hypoxic conditions. 

### HIF-2α was involved in proliferation of epithelial cells under hypoxic conditions induced by RECK silencing

**Figure 3:**
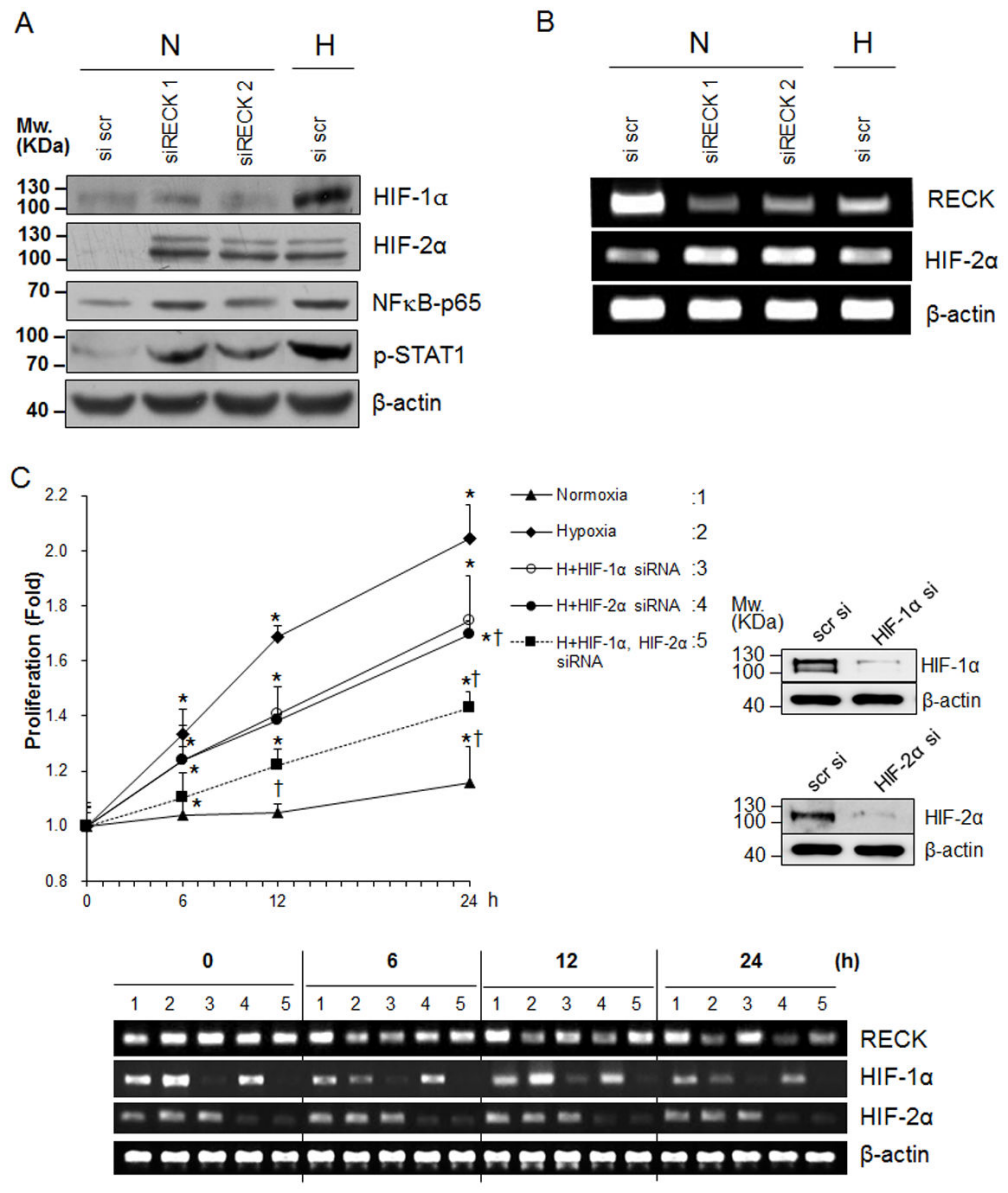
The involvement of HIF-2α in hypoxia-induced RECK silencing-mediated cell proliferation. (**A**) HEK293 cells were transfected with scrambled or RECK siRNA and exposed to normoxic or hypoxic conditions for 24 h. The expressions of HIF-1α HIF-2α, NFκB, and phophorylated-STAT1 were determined by western blotting. β-actin was used as an internal control. (**B**) Using the same samples as in A, the mRNA expressions of RECK and HIF-2α were determined by semi-quantitative RT-PCR. β-actin was used as an internal control. (**C**) HEK293 cells were plated onto 96-well plates and transfected with siHIF-1α and/or siHIF-2α or scrambled siRNA using HiPerFect Transfection reagent (Qiagen). After 24 h, transfected cells were incubated under normoxic or hypoxic conditions for an additional 24 h. At the indicated time points, cell proliferation assays were performed using CCK-8 solution. Data are presented as means ± SDs (n>3). Expression of HIF-1α and HIF-2α was confirmed in each siRNA transfectant by western blot analysis (right panel). RECK, HIF-1α, HIF-2α mRNA expression pattern was confirmed by semiquantitative RT-PCR at indicated time point. β-actin was used as an internal control (lower panel). *, *p*<0.01 significantly different from the normoxic control group at each time point. †, *p*<0.01 significantly different from the hypoxic control group at each time point.

Because HIF-1α and HIF-2α differentially regulate cell cycle progression under hypoxia, we checked the expressions of HIF-1α and HIF-2α under hypoxic and RECK- knockdown conditions, hypoxia increased HIF-1α and HIF-2α protein levels. However, although RECK knockdown induced the upregulation of HIF-2α expression, it did not upregulate HIF-1α (Figure 3A), which suggested RECK is an upstream regulator of HIF-2α but not of HIF-1α. Surprisingly, NFκB and phosphorylated STAT1 was also upregulated by RECK knockdown and hypoxia (Figure 3A). HIF-2α mRNA was upregulated by RECK knockdown and hypoxia (Figure 3B), suggesting RECK regulates HIF-2α at the transcriptional level. 

Hypoxia-induced cell proliferation was blocked in HIF-1α and/or HIF-2α silenced cells from 6 h to 24 h of culture under hypoxic conditions (Figure 3C). We found HIF-1α and HIF-2α siRNAs were effective and confirmed that silencing of HIF-1α inhibited RECK mRNA expression but silencing of HIF-2α did not. Furthermore, the mRNA expressions of HIF-1α and/or HIF-2α under hypoxic conditions were unchanged (Figure 3C, lower panel). 

After 24 h of culture under hypoxic conditions, hypoxia-induced cell proliferation was blocked in HIF-1α and/or HIF-2α silencing cells (Figure 3C). The results may explain that HIF-2α is involved in the cell proliferation mediated by RECK-silencing whereas HIF-1α is directly involved in *RECK* gene silencing at the transcriptional level [8].

### Involvement of EGFR signaling in the HIF-2α expression induced by RECK silencing

**Figure 4:**
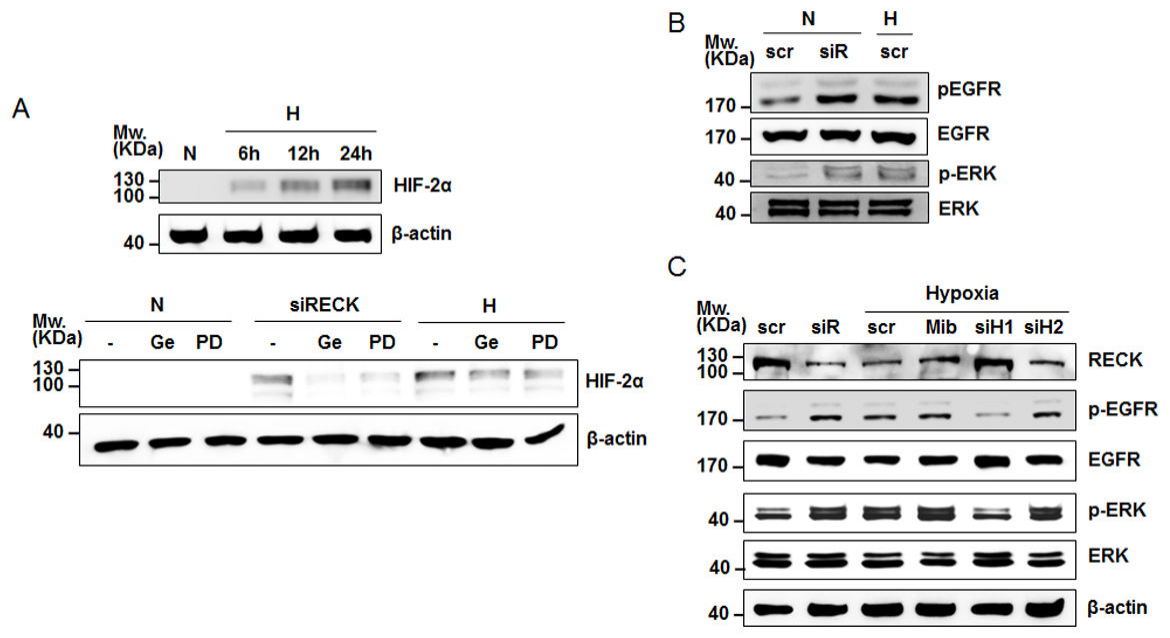
Involvement of the EGFR signaling pathway in HIF-2α expression in RECK-silenced cells. (**A**) HEK293 cells were exposed hypoxia for 6, 12, and 24 h and determined time-dependent HIF-2α expression by western blot analysis (upper panel). HEK293 cells transfected with scrambled si RNA (-) or siRECK were treated with gefitinib (Ge, 1 μM, an EGFR inhibitor) or PD98059 (PD, 50 μM; an ERK MAPK inhibitor) under normoxia (N) or hypoxia (H) for 24 h, and the protein expressions of HIF-2α were determined by western blot analysis. β-actin was used for internal control. (**B**) The activations of EGFR and ERK-MAPK in scrambled (scr) or siRECK (siR) transfected cells exposed to normoxia (N) or hypoxia (H) were determined by western blotting using antibodies against total form or phosphorylated-form of EGFR or ERK. (**C**) The activations of EGFR and ERK and RECK expression in siHIF-1α (siH1) -, siHIF-2α (siH2) -, and MMP inhibitor (Mib)-treated cells under hypoxia (H) were determined by western blot analysis using antibodies against phosphorylated-EGFR and RECK antibody. Scr. Scrambled siRNA.

The fact that RECK inhibits the EGFR signaling pathway [5] made us to examine whether the inhibition of EGFR influences HIF-2α expression induced by RECK silencing. After we confirmed that HIF-2α was upregulated under hypoxia in a time-dependent manner (Figure 4A, upper panel), we treated siRECK-transfected or control cells with gefitinib (Ge, a selective EGFR inhibitor) and PD98059 (PD) under normoxia or hypoxia. Gefitinib suppressed RECK silencing- or hypoxia-induced HIF-2α expression, and PD98059 (a MEK inhibitor) had the same effect as gefitinib on HIF-2α expression (Figure 4A, lower panel). To confirm this result, we checked the activation of EGFR and of its downstream molecule, p42/44 ERK MAPK, which is activated and involved in RECK silencing in hypoxia [21], and found that hypoxia and RECK silencing both activated EGFR and p42/44 ERK MAPK (Figure 4B). In addition, we further investigated whether EGFR activation is regulated by MMP or by the silencing of HIF-1α or HIF-2α. Levels of phosphorylated EGFR and downstream phosphorylated ERK were unchanged by MMP inhibitor (Mib) or siHIF-2α under hypoxia, but changed by siHIF-1α (Figure 4C), indicating the pathway responsible for MMP inhibition differs from required for EGFR activation, and that EGFR activation by RECK silencing is a downstream of HIF-1α and upstream of HIF-2α. These results indicate that the expression of HIF-2α is induced by silencing of RECK via activation of the EGFR signaling pathway. 

### MMP activation was involved in hypoxia-stimulated proliferation

**Figure 5:**
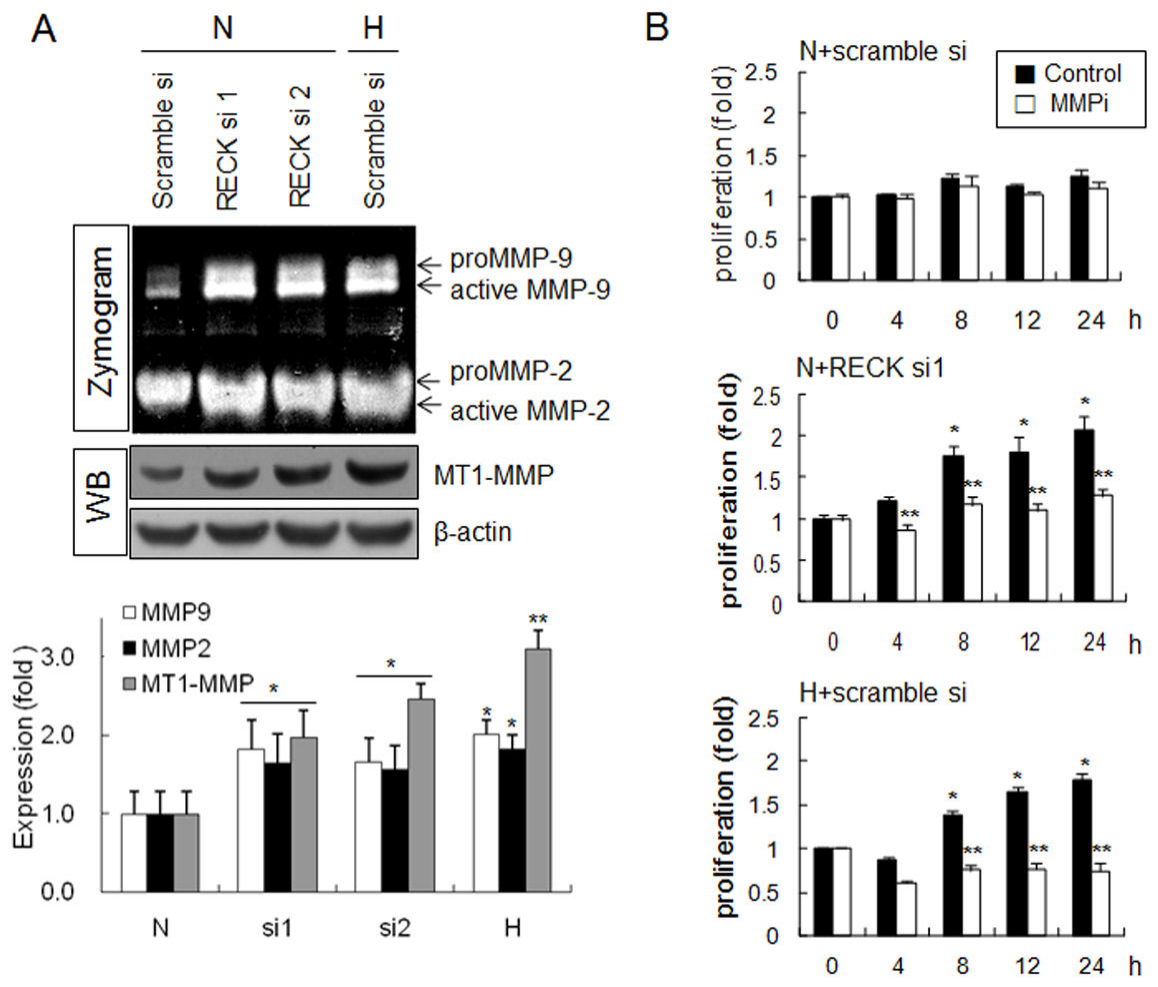
MMP-2/-9 and MT1-MMP were also involved in hypoxia-induced epithelial cell proliferation. (**A**) Cells were transfected with scrambled siRNA or the two types of siRECKs. After 24 h, transfected cells were incubated under normoxic or hypoxic conditions for an additional 24 h. Conditioned media were collected from siRECK transfected (si1 and si2) or hypoxia-exposed HEK 293 cells, and gelatin zymography was performed. Western blotting for MT1-MMP was also performed using protein lysates obtained under each condition. (**B**) HEK293 cells were pretreated with a MMP inhibitor (5 µM) 2 h before normoxic or hypoxic incubation and cell proliferation assays were performed at the indicated times under normoxic (N) or hypoxic (H) conditions. Data are presented as means ± SDs (n=4). *, *p*<0.01 versus the t = 0 control, **, *p*<0.01 versus the vehicle control.

RECK functions as an inhibitor of MMP activation [22,23]. Therefore, we examined MMPs and MT1-MMP by zymography and western blotting under hypoxic conditions. Hypoxia and siRECK transfection significantly induced pro- and active MMP-9 and MMP-2 by zymography, and increased MT1-MMP protein expression levels (Figure 5A). 

Cell proliferation was increased by hypoxia and by RECK siRNA transfection but treatment with the MMP inhibitor decreased cell proliferation induced by hypoxia or siRECK transfection (Figure 5B). These findings suggest that MMP-2 and MMP-9 are involved in the activation of cell proliferation induced by the silencing of RECK under hypoxic conditions, but that the pathway involves differs from that of EGFR-HIF-2α-induced cell proliferation. 

### Silencing of RECK gene promoted *in vitro* and *in vivo* tumor formation

**Figure 6:**
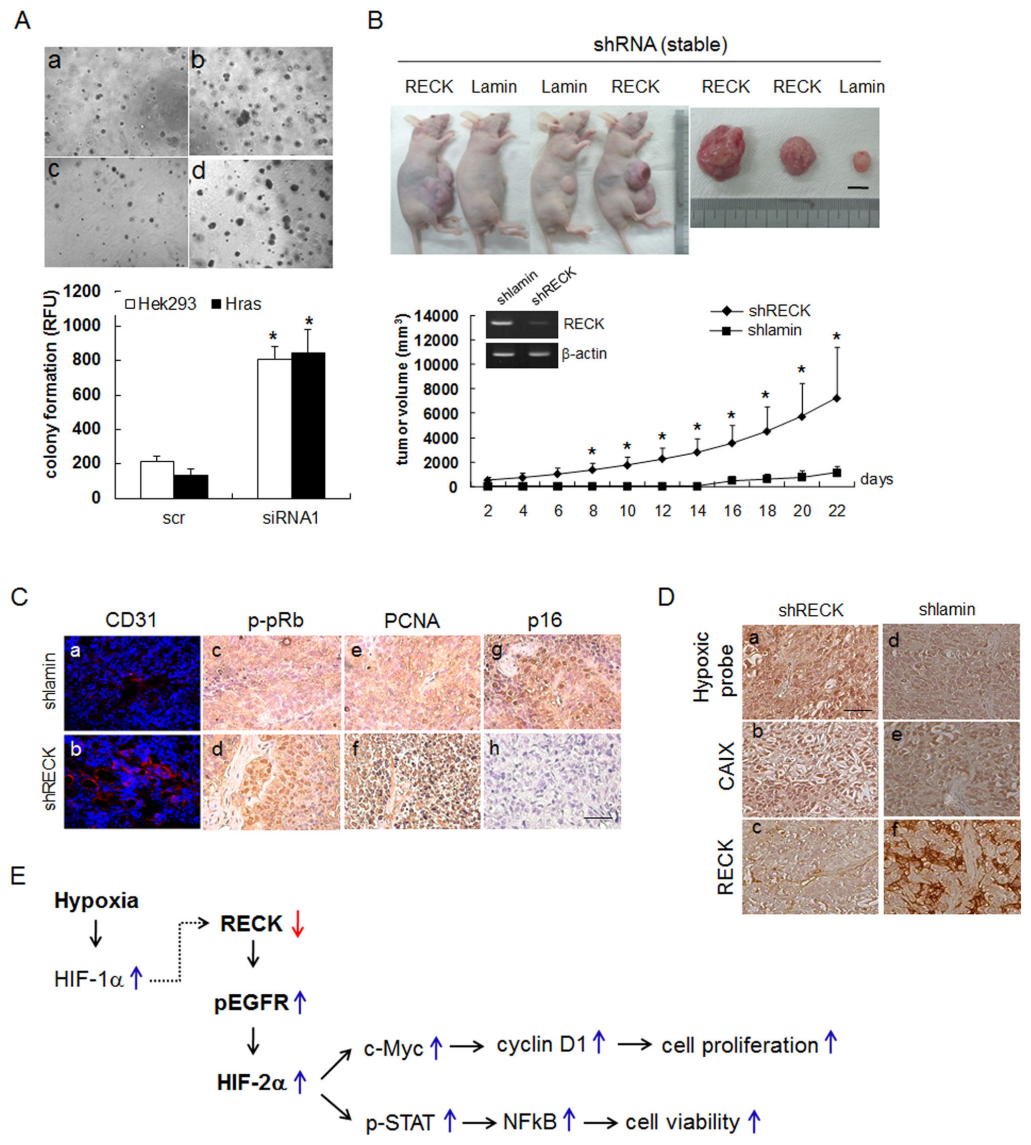
Silencing of *RECK* increased *in* *vitro* soft-agar colony formation and *in* *vivo* tumorigenesis. (**A**) HEK293 cells were transfected with si*RECK* and plated onto agar-coated 96-well plates 24 h after transfection. Colonies were observed one week after seeding (upper panel). Original magnification 40X. Quantitation of colony formation by HEK293 cells transfected with scrambled or RECK siRNA1 one week after seeding. Colony formation was measured using a fluorometer, as described in Materials and Methods. Data presented as means ± SDs (n=4). *, *p*<0.01. (lower panel) (**B**) HEK293 cells stably transfected with shRECK were injected subcutaneously into the flanks of nude mice. One week later, tumor volumes (length x width x height, mm) were measured; animals were sacrificed 22 days after the first measurement. The upper panel shows tumors in sacrificed mice; including the largest and smallest shRECK tumors (RECK) and the largest shlamin tumor (Lamin). RECK expression is shown inside a graph with stable transfectant of shlamin and shRECK with RT-PCR. β-actin was used as an internal control. Scale bar=10 mm. Data are presented as means ± SDs (n=4). *, *p*<0.01. Scale bar=100 µm. (**C**) Immunohistochemical staining analysis with anti-CD31 (a, b), anti-phosphorylated-Rb (c, d), PCNA (e, f) and anti-p16 (g, h) antibodies in the control (shlamin) and shRECK tumors shown in C. Anti-rhodamine (for CD31) and DAB (c-f) were used for visualization. Scale bar=10 µm. (**D**) Immunohistochemical staining analysis with anti-Hypoxic probe-1 (a, d), anti-CA9 (b, e), and anti-RECK (c, f) in xenofraft tumor section of stably transfected shRECK or shlamin (control). Scale bar=100 µm. (**E**) A scheme for the involvement of RECK/EGFR/HIF-2α in normal epithelial proliferation and viability in hypoxia. A dotted line represents findings in reference 8.

To confirm that the downregulation of *RECK* promotes the hyperplastic activity of epithelial cells *in vitro*, we used soft agar colony formation assays [24]. Cells transfected with *RECK* siRNA formed more colonies than those transfected with scrambled siRNA (Figure 6A, a & c vs. b & d), and the fluoroactivities of colony lysates from *RECK* siRNA transfected cells were significantly higher than those of control siRNA transfectants (Figure 6A, graph). 

To explore the role of RECK downregulation in hyperplastic development during early tumorigenesis, we generated stable cells with *RECK*-knockdown. These cells were then injected s.c. into nude mice flank, and tumor masses were measured from 7 days post-injection for 22 days. As early as one week post-injection, nude mice injected with shRECK transfectants bore tumors with greater mass volumes and growth compared to those injected with control cells (Figure 6B). Tumor microvessel density assayed by CD31 immunostaining showed that *RECK* knockdown was associated with highly vascularized tumors (Figure 6C, a-b). Furthermore, levels of p-pRb and of proliferating cell nuclear antigen (PCNA) were markedly higher in shRECK tumors, but p16^INK4A^ expression was completely abolished (Figure 6C, c-h). To confirm the inverse relation between hypoxia and RECK expression in tumors, we examined hypoxic regions in RECK silenced tumors by injecting pimonidazole (a hypoxic probe). Immunohistochemistry showed that hypoxic regions (Figure 5D) and carbonic anhydrase IX (CA9) (a hypoxic marker protein) expressions were greater in RECK silenced tumors (Figure 6D). Taken together, these results suggest that RECK suppression by hypoxia promotes the acquisition of a premalignant hyperplastic phenotype and enhances *in vitro* and *in vivo* tumorigenesis via G1/S phase cell cycle progression through the activation of EGFR and HIF-2α. 

## Discussion

In this study, RECK expression was found to be downregulated by hypoxia in normal epithelial cells and to be involved in the development of the hypoxia-induced hyperplastic phenotype. RECK silencing under hypoxic conditions enhanced cell cycle progression through c-Myc, and silencing of RECK induced EGFR activation and subsequently HIF-2α expression. Colony formation assays and *in vivo* tumor xenograft experiments suggested that the suppression of RECK by hypoxic stress is required to transform normal cells to tumor-like cells by enhancing their proliferative abilities. Therefore, loss of RECK expression by microenvironmental hypoxic stress may stimulate to get a more hyperplastic phenotype in normal epithelial cells and to favor early tumorigenesis events. Our results support previous suggestions that *RECK* functions as a transformation suppressor gene in premalignant and normal tissues [3,23].

We found although hypoxia stabilized HIF-1α and HIF-2α, only HIF-2α was upregulated by silencing of *RECK*, and that knockdown of either HIF-1α or HIF-2α recovered hypoxia-induced cell proliferation (Figure 3). Because HIF-1α participates in *RECK* downregulation at the transcription level [8], we can speculate that HIF-1α and HIF-2α are upstream and downstream regulators of RECK, respectively under hypoxic conditions. But HIF-2α is directly involved in RECK-silencing-mediated cell proliferation.

HIF-2α and c-Myc may collaborate to increase cell proliferation under hypoxic conditions and in RECK silencing cells. HIF-1α and HIF-2α have opposing effects on c-Myc interaction and cell proliferation [12]. Under hypoxic conditions, HIF-1α and HIF-2α are competent to occupy c-Myc. HIF-1α participates in gene silencing at *RECK* promoter in accompany with HDAC1 [8], and in the donating of c-Myc to HIF-2α rather than binding to c-Myc to cause hypoxia-induced cell proliferation. Since HIF-1α and -2α are expressed in a cell-type specific manner [25], hypoxic conditions might have different effects on cell proliferation depending on the availabilities of HIF-1α and HIF-2α in a variety of cells. In a recent study, HIF-2α was present in a stem cell population but HIF-1α was present in both stem and non-stem cell populations and was only stabilized under more severe hypoxic conditions [26]. HIF-2α transcriptionally activates the Oct4 gene, a stem cell marker [27]. A significant evidence supports our hypothesis that HIF-2α alters the basic genetic activity of normal transformed cells to a more stem-like hyperplastic phenotype in a manner that depends on the fluctuating state of oxygen availability under chronic pathophysiological conditions [28]. Furthermore, a switch of hypoxic response from HIF-1 to HIF-2-depedent gene expression results in high aggressive tumor through the promotion of stem cell characteristics [29]. In CD133+ glioblastoma cells, the expressions of MMPs and RECK were induced by miR-125b, and the authors suggested that the functional inhibition of RECK increases cancer stem cell function [30]. Our findings suggest that RECK is likely to be an important molecule for regulation of switch from HIF-1α- to HIF-2α- dependent transcription. 

Although it has been shown that hypoxia induces cell cycle arrest but not proliferation [31,32], exposure time and degree of hypoxia can also affect cell cycle arrest/survival decisions. For example, longer and more severe hypoxia induces cell cycle arrest and apoptosis to a greater extent than shorter and milder exposure [32]. Hypoxia and the silencing of RECK by siRNA increased the protein expressions of c-Myc, cyclin D1, cyclin A, and p-pRb, but decreased those of p21^cip1^, p27^kip1^ and p16^ink4A^, indicating that the G1/S transition is a key regulation point of the hypoxia-induced proliferation of epithelial cells. These results are consistent with the previous findings that RECK depletion stimulates cell proliferation during successive cell cultivations, and that this is correlated with diminished expressions of p21^cip1^, p53, and p19^ink4D^ in mouse MEFs [5]. 

In cancer cells, the repression of RECK by hypoxia also influenced cell proliferation and enhanced migration or invasion [21,23,33]. Yoshida et al. found that the inhibition of cancer cell proliferation is mediated by S-phase kinase-associated protein 2 (SKP2) targeting p27^kip1^ [34], which suggests that different molecules might be involved in the suppression of cell growth by RECK in a cell-specific manner. Thus, the downregulation of RECK and the stabilization of HIF-2α by hypoxia may contribute to the development of the hyperplastic phenotype in premalignant cells by increasing the expressions of genes involved in cell cycle progression. The activation of HIF-2α in cancer cells increases the expression of EGFR [35] in response to hypoxia [36]. In a study using EGFR inhibitor, it was suggested that HIF-1α might be a downstream regulator of EGFR [37]. However, our findings suggest that EGFR might be involved in the upregulation of HIF-2α in RECK-silenced cells and in cells under hypoxic conditions (Figure 4B). Importantly, findings in VHL-/- cancer cells demonstrated that the shRNA-mediated inhibition of EGFR was sufficient to abolish HIF-2α-induced spheroid formation in a three-dimensional tumorigenic assay [38], which suggests that EGFR is required for HIF-2α-mediated tumorigenesis. Furthermore, the inactivation of EGFR can be achieved by the restoration of RECK [5]. Therefore, it is suggested that the RECK-EGFR-HIF-2α axis is a critical determinant of the hyperplastic phenotype in hypoxic cells. 

Because an MMP inhibitor decreased proliferation rate induced by hypoxia, MMP inhibition by hypoxia-induced RECK silencing involves a different pathway from involving EGFR-HIF-2α during the inhibition of cell proliferation. As MMPs are also involved in cell proliferation [39], MMP inhibition by RECK downregulation also suppresses cell proliferation induced by hypoxia. These findings suggest that these two pathways work together to increase cell proliferation.

Interestingly, it has been previously reported that the activation of NFκB in intestinal epithelial cells and macrophages results in the inductions of anti-apoptotic genes and cytokines that increase the survival and proliferation of premalignant intestinal epithelial cells [40]. In our findings, activation of NFκB and STAT1 both by hypoxia and RECK silencing may also participate the cell proliferation and survival.

The restoration of *RECK* and the destabilization of HIF-2α could perhaps be adopted as a means of preventing the early transformation of hyperplastic cells in premalignant lesions under hypoxic conditions. However, further investigations are needed to characterize the mechanism by which RECK regulates c-Myc or HIF-2α during transformation to the hyperplastic phenotype and during the cell cycle progression of normal cells. The observation that hypoxia can cause *RECK* silencing in normal cells is important, as it could be a novel mechanism for reducing tumor suppressor expression under low oxygen tension-dependent stress, which characteristic of certain pathological conditions. 
